# Risks associated with magnetic resonance imaging and cervical collar in comatose, blunt trauma patients with negative comprehensive cervical spine computed tomography and no apparent spinal deficit

**DOI:** 10.1186/cc6957

**Published:** 2008-07-14

**Authors:** C Michael Dunham, Brian P Brocker, B David Collier, David J Gemmel

**Affiliations:** 1 Trauma/Critical Services, St. Elizabeth Health Center, Level I Trauma Center, Belmont Avenue, Youngstown, Ohio 44501, USA; 2 Neurosurgery, St. Elizabeth Health Center, Level I Trauma Center, Belmont Avenue, Youngstown, Ohio 44501, USA; 3 Radiology, St. Elizabeth Health Center, Level I Trauma Center, Belmont Avenue, Youngstown, Ohio 44501, USA; 4 Medical Research, St. Elizabeth Health Center, Level I Trauma Center, Belmont Avenue, Youngstown, Ohio 44501, USA

## Abstract

**Introduction:**

In blunt trauma, comatose patients (Glasgow Coma Scale score 3 to 8) with a negative comprehensive cervical spine (CS) computed tomography assessment and no apparent spinal deficit, CS clearance strategies (magnetic resonance imaging [MRI] and prolonged cervical collar use) are controversial.

**Methods:**

We conducted a literature review to delineate risks for coma, CS instability, prolonged cervical collar use, and CS MRI.

**Results:**

Based on our search of the literature, the numbers of functional survivor patients among those who had sustained blunt trauma were as follows: 350 per 1,000 comatose unstable patients (increased intracranial pressure [ICP], hypotension, hypoxia, or early ventilator-associated pneumonia); 150 per 1,000 comatose high-risk patients (age > 45 years or Glasgow Coma Scale score 3 to 5); and 600 per 1,000 comatose stable patients (not unstable or high risk). Risk probabilities for adverse events among unstable, high-risk, and stable patients were as follows: 2.5% for CS instability; 26.2% for increased intensive care unit complications with prolonged cervical collar use; 9.3% to 14.6% for secondary brain injury with MRI transportation; and 20.6% for aspiration during MRI scanning (supine position). Additional risk probabilities for adverse events among unstable patients were as follows: 35.8% for increased ICP with cervical collar; and 72.1% for increased ICP during MRI scan (supine position).

**Conclusion:**

Blunt trauma coma functional survivor (independent living) rates are alarming. When a comprehensive CS computed tomography evaluation is negative and there is no apparent spinal deficit, CS instability is unlikely (2.5%). Secondary brain injury from the cervical collar or MRI is more probable than CS instability and jeopardizes cerebral recovery. Brain injury severity, probability of CS instability, cervical collar risk, and MRI risk assessments are essential when deciding whether CS MRI is appropriate and for determining the timing of cervical collar removal.

## Introduction

Blunt trauma patients with coma (Glasgow Coma Scale [GCS] score 3 to 8) are at increased risk for cervical spine (CS) injury [1,2]. Reported CS injury rates are 10.5% to 14.0% [3,4]. To enhance detection of CS injuries in comatose patients, several authors have recommended a CS computed tomography (CT) scan with the first brain CT [3,5,6].

Spine surgical consultation and magnetic resonance imaging (MRI) are usual when fracture, malalignment, or prevertebral swelling is identified during the CT evaluation or a spinal deficit is apparent. However, when there is no apparent spinal deficit or CT evidence for acute injury, the need for ancillary imaging with coma is controversial [7-11]. There is concern that a comatose patient may have an isolated ligamentous injury and spinal column instability, even though the reported rate is low [12]. To identify isolated ligamentous injury, some investigators recommend CS MRI or dynamic fluoroscopy [12], but others have found that dynamic fluoroscopy is of limited value [11,13,14]. Several authors have questioned the use of dynamic fluoroscopy or have abandoned the procedure because of inadequate CS imaging, safety concerns, or expense [14-17].

There are four common cervical collar removal choices in comatose, blunt trauma patients without CS fracture, malalignment, prevertebral swelling, or apparent spinal deficit. One option is early cervical collar removal without CS MRI. Another choice is cervical collar removal after an early MRI (within 72 hours) reveals no CS instability. A third option is late cervical collar removal without MRI. Specifically, this is removal of the cervical collar if there is no neck pain or tenderness, once awareness improves. If there is neck pain or tenderness, then spine surgical consultation or MRI should be obtained. The fourth choice is cervical collar removal after a delayed MRI reveals no CS instability. The pros and cons of each choice are controversial.

Because these patients have severe brain injury, the risk for secondary brain injury associated with each cervical collar removal option needs exploration. A risk and benefit assessment should include estimates of the risk for secondary brain injury with each choice and of the probability that CS instability exists.

The primary purpose of this study is to estimate the risks associated with early cervical collar removal without MRI in blunt trauma comatose patients, when comprehensive CS CT imaging reveals no sign of acute injury and there is no apparent spinal deficit. Another goal is to describe secondary brain injury risks associated with the CS collar and MRI scanning. An additional objective is to explore the potential impact of CS collar and MRI risks on brain injury outcomes (functional survival, severe disability, and death).

## Materials and methods

A literature review was undertaken to identify risks associated with early cervical collar removal without MRI, late cervical collar removal, transportation to the MRI scanner, MRI scanning, and severe brain injury. For each risk search, the title and abstracts were assessed to determine article relevance. The bibliography of each pertinent article was reviewed for additional germane evidence.

### Defined terms

The basis for describing outcomes in this manuscript is the Glasgow Outcome Scale. The components are dead, vegetative state, severe disability, moderate disability, and good recovery. We define 'functional survival' as a moderate disability or good recovery outcome. These patients are capable of independent activities of daily living. We define 'severe disability' as a vegetative state or severe disability outcome. These patients are incapable of independent activities of daily living. Using literature-based data, we have created a traumatic, comatose patient classification that implies specific patient care needs and prognosis (see Risk for death or severe disability in severe brain injury in the Results section, below). 'Unstable patients' are those with intracranial hypertension, systemic hypotension, hypoxemia, or early ventilator-associated pneumonia (VAP). 'High-risk patients' are those with admission GCS scores of 3 to 5 or age above 45 years. 'Stable patients' are not in the unstable or high-risk categories (no intracranial hypertension, no hypotension, no hypoxemia, no early VAP, admission GCS score 6 to 8, and age 15 to 45 years).

### Risks associated with early cervical collar removal without MRI

A comprehensive literature review was undertaken to identify studies of obtunded or comatose patients with no apparent spinal deficit and negative CS bony imaging assessments and to calculate rates of CS instability in these patients. Because studies of obtunded patients include those with coma, they were considered relevant. Studies were classified according to mental status: coma or obtunded. Investigations were categorized as coma patient studies if the inclusion criteria were specific (coma, severe brain injury, GCS score ≤ 8, or unconscious) or the Results section documented a group GCS score of 8 or less. Investigations were classified as obtunded if the inclusion criteria were specific (obtunded or unexaminable and the group GCS score was >8 or not stated). PubMed was explored for articles published during the past 10 years that discussed or presented relevant data. The search terms were as follows: 'cervical vertebrae' (medical subject heading [MeSH]) AND 'acute brain injury' (MeSH), 'coma' (MeSH), or 'obtunded' (title).

Manuscripts were selected using several criteria. The patients were obtunded or comatose and had sustained an injury of blunt trauma mechanism. The patients underwent comprehensive CS bony examination without evidence of acute injury (no fracture, no malalignment, and no prevertebral swelling). Comprehensive CS bony examination included plain radiographs with selected CT scanning (nonvisualized or suspicious areas) or comprehensive CT scanning (routine occiput through T-1 with axial images and sagittal and coronal reformats). All patients underwent a CS confirmatory examination (dynamic fluoroscopy, MRI, or subsequent neck examination). Studies were included if there was an indication that patients with spinal cord injury were excluded. Spinal column instability was implied if there was a need for a halo vest, surgical stabilization, or maintenance of a cervical collar for 4 to 6 weeks. Studies were categorized as prospective or retrospective.

### Risks associated with late cervical collar removal

The literature was searched for evidence that a cervical collar can raise intracranial pressure (ICP). Three search strategies were performed in PubMed. One approach was to use the search terms 'cervical vertebrae' (MeSH) and 'intracranial hypertension' (MeSH). Another method was to use the search terms 'cervical vertebrae' (MeSH) and 'intracranial pressure' (MESH). The third procedure was to use the search terms 'collar' (title) and 'intracranial pressure' (title). When reviewing the articles describing the rate of cervical spine instability in blunt trauma comatose patients with a negative CS CT scan, one article was identified that cited the intensive care unit (ICU) complication rate with and without early cervical collar removal [18].

### Risks associated with transportation to the MRI scanner

A PubMed search was conducted to ascertain the potential risks associated with out-of-ICU transportation. The MeSH headings 'intensive care units' and 'transportation' were utilized.

### Risks associated with MRI scanning

Because brain injured patients are placed in the supine position during MRI scanning, a comparison of risks by position was undertaken. A PubMed literature search was performed to assess the ICP effect of lowering the head with severe brain injury. MeSH terms were 'intracranial pressure'; 'head injuries' or 'brain injuries'; and 'posture' or 'supine position'. Study results were evaluated when ICP was documented in the supine and head-up positions in comatose, trauma patients.

Also, because of supine positioning during MRI scanning, a review was undertaken to assess the risk for aspiration and VAP when the patient's head is lowered during mechanical ventilation. A PubMed literature search was performed using the following MeSH terms: 'pneumonia', 'posture', and 'respiration, artificial'.

### Risk for death or severe disability in severe brain injury

The outcome after severe brain injury is contingent on factors other than CS injury. To evaluate the impact of those traits on death and severe disability, a PubMed search was performed. The MeSH terms 'brain injuries' and 'outcome assessment' were used to initiate the literature examination. Brain injury outcomes described in this report are a dichotomization of the Glasgow Outcome Scale. 'Functional survival' patients are those with moderate disability or good recovery (capable of independent activities of daily living). 'Death or severe disability' patients are those who die or survive, where the survivors are in a vegetative state or have severe disability (incapable of independent activities of daily living).

## Results

### Risks associated with early cervical collar removal without MRI

Table 1 summarizes the studies in which plain radiographs and supplementary CT (to image suspicious or nonvisualized areas) revealed no sign of acute bony spinal column injury. The investigators were D'Alise [19], Davis [13], Hogan [20], and Padayachee [21] and their colleagues. Some studies included patients with CS bony injuries. However, data presentation is such that computation of CS instability in the subset without bony injury is possible. Table 2 shows the studies in which comprehensive CS CT revealed no sign of acute bony spinal column injury. Researchers were Adams [22], Brohi [3], Como [23], Ghanta [24], Menaker [25], Sarani [26], Schuster [27], Stassen [28], Stelfox [18] and Widder [29], and their colleagues. In 10 of the 14 studies [13,19,20,22-27,29] the authors explicitly stated or indicated by patient grouping that patients with apparent spinal deficit were excluded. Three of the other reports [3,18,21] do not describe any patients with spinal cord injury. Only one study [28] describes a few patients with spinal cord injury. The CS column instability rates for the 14 studies are presented in Table 3. When bony comprehensive CS imaging shows no evidence of acute injury and there is no apparent spinal deficit, the estimated risk for spinal column instability in comatose or obtunded patients is 2.5% (25 in 1,000).

**Table 1:** Cervical spine instability studies in obtunded/comatose blunt trauma patients with no apparent spinal deficit and negative cervical spine plain radiographs with supplementary CT scans

Study	Prospective	LI/instability assessment	Mental status
D'Alise *et al*. (1999) [19]	Yes	MRI in all patients; flexion/extension radiographs when MRI was negative (83%)	Obtunded
Davis *et al*. (2001) [13]	Yes	DF in all patients	Coma
Hogan *et al*. (2005) [20]	No	MRI in all patients	Obtunded
Padayachee *et al*. (2006) [21]	Yes	MRI in some patients; DF in all patients (adequate in 97%)	Coma

CT, computed tomography; DF, dynamic fluoroscopy; LI, ligamentous injury; MRI, magnetic resonance imaging.

**Table 2:** Cervical spine instability studies in obtunded/comatose blunt trauma patients with no apparent spinal deficit and negative comprehensive cervical spine CT scans

Study	Prospective	LI/instability assessment	Mental status
Adams, *et al*. (2006) [22]	No	MRI in all patients	Obtunded
Brohi, *et al*. (2005) [3]	Yes	MRI and/or clinical follow-up in all patients	Coma
Como, *et al*. (2007) [23]	Yes	MRI in all patients	Coma
Ghanta, *et al*. (2002) [24]	No	MRI in all patients	Obtunded
Menaker, *et al*. (2008) [25]	No	MRI in all patients	Obtunded
Sarani, *et al*. (2007) [26]	No	MRI in all patients	Obtunded
Schuster, *et al*. (2005) [27]	No	MRI in all patients	Coma
Stassen, *et al*. (2006) [28]	No	MRI in all patients	Coma
Stelfox, *et al*. (2007) [18]	Yes	MRI, flexion/extension radiographs, and/or clinical follow-up in all patients	Obtunded
Widder *et al*. (2004) [29]	Yes	Clinical follow-up in-hospital and post-discharge	Coma

CT, computed tomography; LI, ligamentous injury; MRI, magnetic resonance imaging.

**Table 3:** Cervical spine instability rates in obtunded/comatose blunt trauma patients with no apparent spinal deficit and negative comprehensive bony spinal column imaging

Study	Total	Collar (4 to 6 weeks)	Halo/ORIF	Either treatment
Adams, *et al*. (2006) [22]	20	--	0	0
Brohi, *et al*. (2005) [3]	381	--	0	0
Como, *et al*. (2007) [23]	115	0	0	0
Davis *et al*. (2001) [13]	300	0	1	1
D'Alise *et al*. (1999) [19]	108	17	1	18
Ghanta, *et al*. (2002) [24]	46	2	0	2
Hogan *et al*. (2005) [20]	366	--	0	0
Menaker, *et al*. (2008) [25]	203	15	2	17
Padayachee *et al*. (2006) [21]	276	--	0	0
Sarani, *et al*. (2007) [26]	46	4	0	4
Schuster, *et al*. (2005) [27]	12	0	0	0
Stassen, *et al*. (2006) [28]	44	13	0	13
Stelfox, *et al*. (2007) [18]	215	--	0	0
Widder *et al*. (2004) [29]	84	--	0	0
				
Total	2,216	51	4	55

This table is an amalgamation of of the studies included in Tables 1 and 2. Cervical collar, 2.3% (1.8% to 3.0%); halo/open-reduction with internal fixation (ORIF), 0.2% (0.1% to 0.5%); either treatment, 2.5% (1.9% to 3.2%).

### Risks associated with use of cervical collar

Mobbs [30], Davies [31], and Hunt [32] and their colleagues documented that cervical collars are associated with increased ICP in the setting of traumatic brain injury. The three studies demonstrated that ICP increases by about 5 mmHg with cervical collar application (all values mmHg): 25.8 versus 20.5 (*P *< 0.05) [30], 18.4 versus 13.3 (*P *< 0.001) [31], and 18.8 versus 14.1 (*P *< 0.0001) [32]. Individual patient ICP data with and without cervical collar application were presented in two reports (n = 29) [30,31]. With cervical collar application, ICP increased 5 mmHg in 53.6% (95% confidence interval [CI] = 35.8% to 70.5%). In patients with a pre-collar ICP of 15 mmHg or less, the rate post-collar ICP of 20 mmHg or greater was 27.8% (95% CI = 12.5% to 50.9%). A recent investigation demonstrated that ICU complications are associated with prolonged cervical collar use in obtunded trauma patients with negative CS CT [18]. The ICU complication rates for late collar removal and early collar removal were 63.5% and 37.3%, respectively (*P *= 0.01). The complication rate difference was 26.2%.

### Risks associated with transportation to the MRI scanner

Physiologic instability and secondary brain injury are common in neurosurgical and traumatic brain injury ICU patients transported for diagnostic imaging [33-39]. Gunnarsson and coworkers [33] compared the rate of secondary brain injury events (cardiovascular instability, increased ICP, hypoxia, or seizures) in neurosurgical ICU patients undergoing CT scanning in the radiology suite or in the ICU. Secondary brain injury events were more common in patients transported to the radiology suite (*P *= 0.004). Secondary brain injury events occurred in 25.0% (95% CI = 14.6% to 39.4%) of unstable or high-risk patients (cardiovascular or respiratory instability or an ICP = 20 mmHg) transported to the radiology suite. Secondary brain injury events developed in 17.8% (95% CI = 9.3% to 31.3%) of stable patients (physiologic stability but requiring an ICP device or undergoing mechanical ventilation) traveling out of the ICU. In another study, Bekar and colleagues [38] showed that ICP substantially increases during out-of-ICU transportation (*P *< 0.01). Additionally, Andrews and coworkers [39] documented that 51% of brain-injured patients transferred from the ICU for diagnostic imaging or operative intervention developed complications, including hypoxia, hypotension, and intracranial hypertension.

### Risks associated with MRI scanning

Six studies documenting 223 patient observations revealed that ICP increases with lowering the head of the bed (*P *< 0.05) [40-45]. Using mean data from the six investigations, the increase in ICP varies from 3.4 to 8.8 mmHg. However, in one study [46], including 30 observations, ICP increased with elevation of the head of the bed; the authors of that report believe that any patient movement increases ICP. In studies commenting on individual patients, ICP increased with lowering the head of the bed in 79.1% (95% CI = 72.1% to 84.7%) of 158 observations [40-44,46,47]. In the other 33 patients, ICP decreased or was without change. Three studies [40,41,45] described the effect of lowering the head of the bed when the head elevated ICP is under 20 mmHg. In the studies by Ng and colleagues [40] and Winkelman [41], head lowering did not increase ICP above 20 mmHg in any of 29 patients (0%; 95% CI = 0% to 11.7%). In the third study, Meixensberger and colleagues [45] demonstrated, in 73 observations, that with head lowering the mean ICP remains less than 20 mmHg. However, it is clear from a figure presented in their report that a few patients had an ICP above 20 mmHg. There was a 72.1% likelihood (95% lower confidence limit) that lowering the head of the bed would increase ICP. It is likely that the ICP increase about 5 mmHg.

VAP occurs in 41% to 60% of severely brain-injured patients [48-52]. According to several review articles [53-56], the evidence supports supine positioning during mechanical ventilation as a risk factor for aspiration and VAP. In a relevant study [57], numerous risk factors for ICU nosocomial infection were investigated in 944 patients. Using multivariate analysis to adjust for confounding variables, the head of the bed in a horizontal position was found to carry the greatest risk for ICU nosocomial infection (hazard ratio = 5.95). The authors' inference was that 'the horizontal position of the head of the bed should be avoided totally'. In a randomized, crossover trial of mechanically ventilated patients, Torres and coworkers [58] instilled radioactive material into the stomach via gastric tube. Mean radioactive counts in endobronchial secretions were higher in the supine position, when compared with semi-recumbent posture. Within 30 and 60 minutes of instillation, endobronchial radioactive counts were greater in supine patients. The investigators concluded that supine position and time spent in this posture are risk factors for aspiration of gastric contents, despite inflation of the endotracheal tube cuff.

Supine position during mechanical ventilation was found to be a risk factor for VAP [59]. The VAP rates for semi-recumbent and supine patients were 5.1% and 23.4% (*P *= 0.018). The VAP rate difference between supine and semi-recumbent positioning was 18.3% (relative risk = 4.56). A prospective study [60] indicated that supine head positioning during the first 24 hours of mechanical ventilation has an independent association with VAP. The supine patient Acute Physiology and Chronic Health Evaluation II score was higher, but it was not clinically different (16.7 versus 14.3). The VAP rates for semi-recumbent and supine patients were 11.2% and 34.0%, respectively (*P *< 0.001). The VAP rate difference between supine and semi-recumbent positioning was 22.8% (relative risk = 3.0). Combining the studies conducted by Drakulovic and coworker [59] and Kollef [60], the estimated VAP rate difference between supine and semi-recumbent positioning is 20.6%.

### Risk for death or severe disability in severe brain injury

With severe brain injury, Marshall and coworkers [61] found the death or severe disability rate to be 58.6% and the functional survival (capable of independent activities of daily living) rate to be 41.4%.

Intracranial hypertension occurred in 48.5% to 68.1% of severe brain injury patients [62-64] and was associated with increased death or severe disability [62-66]. With intracranial hypertension, the death or severe disability rate was 71.2% [64]. Systemic hypotension also exhibited an association with severe brain injury mortality [62,63,65,67]. The death or severe disability rate with hypotension was found to be 64.4% [67]. In addition, hypoxia was found to have an association with death and severe disability [63,64]. The death or severe disability rate with hypoxia was 66.3% [64]. VAP occurred in 41% to 60% of severe brain injury patients [48-52]. Early-onset VAP, which occurred during the first 3 to 4 days after injury, accounted for a large percentage of VAP events with severe brain injury [49,51,68,69] and was associated with hypoxia [70]. VAP can increase severe brain injury ICU complications, as manifested by increased ventilator and ICU days [49,52,71,72]. VAP was also associated with increased severe brain injury mortality [72] and was a significant independent predictor of death or severe disability [48]. The death or severe disability rate for the total population was 56.4% and, although the rate for patients with VAP is not given in the report, the estimated rate is 60% to 65% [48]. The estimated functional survival rate for unstable patients (intracranial hypertension, systemic hypotension, hypoxemia, or VAP) is 35%.

The death or severe disability rates with admission GCS score 3 to 5 or aged above 45 years were 83.8% [61] and 86.0% [73]. The projected functional survival rate for high-risk patients (admission GCS score 3 to 5 or age >45 years) is 15%.

Stable patient functional survival rates [are] as follows: 62.4% with no intracranial hypertension [64]; 51.1% with no hypotension or hypoxia [67]; 64.1% with admission GCS score 6 to 8 [61]; and 47.0% for age 15 to 45 years [73]. The estimated functional survival rate for stable patients (no intracranial hypertension, no hypotension, no hypoxemia, no VAP, admission GCS score 6 to 8, and age 15 to 45 years) is 60%.

### Risks and benefits of cervical collar removal options

Risk estimations are summarized in Table 4.

**Table 4:** Estimated collar management risks in functional survivors with negative CS CT and no apparent spinal deficit

Collar management options	Risk	Risk rate	Patients at risk
Unstable patients (350 functional survivors^a^)			
Early collar removal (no MRI)	CS instability	2.5%	9
Cervical collar	↑ ICP (~5 mmHg)	35.8%	125
Prolonged collar use	↑ ICU complications	26.2%	92
MRI (transportation)	↑ ICP, ↓ BP, hypoxia	14.6%	51
MRI (head down)	↑ ICP (~5 mmHg)	72.1%	252
MRI (head down)	Aspiration or VAP	20.6%	72
High-risk patients (150 functional survivors^a^)			
Early collar removal (no MRI)	CS instability	2.5%	4
Prolonged collar use	↑ ICU complications	26.2%	39
MRI (transportation)	↑ ICP, ↓ BP, hypoxia	14.6%	22
MRI (head down)	Aspiration or VAP	20.6%	31
Stable patients (600 functional survivors^a^)			
Early collar removal (no MRI)	CS instability	2.5%	15
Prolonged collar use	↑ ICU complications	26.2%	157
MRI (transportation)	↑ ICP, ↓ BP, hypoxia	9.3%	56
MRI (head down)	Aspiration or VAP	20.6%	124

^a^'Functional survivors' are the expected functional survivors per 1,000 patients. BP, blood pressure; CS, cervical spine; CT, computed tomography; ICP, intracranial pressure; ICU, intensive care unit; MRI, magnetic resonance imaging; VAP, ventilator-associated pneumonia.

#### Unstable patients

We found the functional survivor probability among unstable patients (intracranial hypertension, systemic hypotension, hypoxia, or early VAP) to be approximately 35% (350 in 1,000 comatose unstable patients).

The literature-based estimate for probability of CS instability with a negative CS CT and no apparent spinal deficit is 2.5%. Thus, nine patients with expected functional survival are at risk for death or severe disability with cervical collar removal without MRI (2.5% of 350 patients).

The literature-based estimate for probability that cervical collar application will increase ICP by about 5 mmHg is 35.8% (95% lower confidence limit). Thus, 125 patients with expected functional survival are at risk for death or severe disability with cervical collar application (35.8% of 350 patients).

The literature-based estimate for the probability that ICU complications will increase because of prolonged cervical collar use is 26.2%. Thus, 92 patients with expected functional survival are at additional risk for ICU complications with prolonged cervical collar application (26.2% of 350 patients).

The literature-based estimate for probability of secondary brain injury with out-of-ICU transportation is 14.6% (95% lower confidence limit). Thus, 51 patients with expected functional survival are at risk for death or severe disability with an out-of-ICU transportation (14.6% of 350 patients).

The literature-based estimate for probability that ICP will increase about 5 mmHg because of supine positioning during MRI is 72.1%. Thus, 252 patients with expected functional survival are at risk for an increase in ICP of about 5 mmHg with supine positioning (72.1% of 350 patients).

The literature-based estimate for probability that aspiration or VAP will occur because of supine positioning during MRI is 20.6%. Thus, 72 patients with expected functional survival are at additional risk for aspiration or VAP with supine positioning during MRI (20.6% of 350 patients).

#### High-risk patients

The functional survivor probability in high-risk patients (admission GCS score 3 to 5 or age >45 years) is approximately 15% (150 in 1,000 comatose high-risk patients).

The literature-based estimate for probability of CS instability with a negative CS CT and no apparent spinal deficit is 2.5%. Thus, four patients with expected functional survival are at risk for death or severe disability with cervical collar removal without MRI (2.5% of 150 patients).

The literature-based estimate for probability that ICU complications will increase because of prolonged cervical collar use is 26.2%. Thus, 39 patients with expected functional survival are at additional risk for ICU complications with prolonged cervical collar application (26.2% of 150 patients).

The literature-based estimate for probability of secondary brain injury with an out-of-ICU transportation is 14.6% (95% lower confidence limit). Thus, 22 patients with expected functional survival are at risk for death or severe disability with an out-of-ICU transportation (14.6% of 150 patients).

The literature-based estimate for probability that aspiration or VAP will occur because of supine positioning during MRI is 20.6%. Thus, 31 patients with expected functional survival are at additional risk for aspiration or VAP with supine positioning during MRI (20.6% of 150 patients).

#### Stable patients

The functional survivor probability in stable patients (admission GCS score 6 to 8, age 15 to 45 years, and without intracranial hypertension, hypotension, hypoxia, or early VAP) is approximately 60% (600 in 1,000 comatose stable patients).

The literature-based estimate of probability of CS instability with a negative CS CT and no apparent spinal deficit is 2.5%. Thus, 15 patients with expected functional survival are at risk for death or severe disability with cervical collar removal without MRI (2.5% of 600 patients).

The literature-based estimate for probability that ICU complications will increase because of prolonged cervical collar use is 26.2%. Thus, 157 patients with expected functional survival are at additional risk for ICU complications with prolonged cervical collar application (26.2% of 600 patients).

The literature-based estimate for probability of secondary brain injury with an out-of-ICU transportation in stable patients is 9.3% (95% lower confidence limit). Thus, 56 patients with expected functional survival are at risk for death or severe disability with an out-of-ICU transportation (9.3% of 600 patients).

The literature-based estimate for probability that aspiration or VAP will occur because of supine positioning during MRI is 20.6%. Thus, 124 patients with expected functional survival are at an additional risk for aspiration or VAP with supine positioning during MRI (20.6% of 600 patients).

## Discussion

The projected number of functional survivors (capable of independent activities of daily living) per 1,000 comatose unstable patients (intracranial hypertension, hypotension, hypoxia, or early VAP) is 350 (35%). The estimated numbers of functional survivors per 1,000 comatose high-risk patients (age >45 years or admission GCS score 3 to 5) and per 1,000 comatose stable patients (not unstable or high risk) are 150 (15%) and 600 (60%), respectively. Because CS injury increases with coma and can affect functional survival, a comprehensive CS CT (occiput to T-1 axial views, with sagittal and coronal reformats) with the initial brain scan is appropriate. This review suggests that comatose blunt trauma patients with no apparent spinal deficit and no acute injury on comprehensive CS CT imaging (no fracture, malalignment, or prevertebral swelling) have a 2.5% probability of CS instability. However, secondary brain injury risk estimates with the cervical collar and MRI scanning are substantially greater. It is imperative to remain focused on the principal strategy for maximizing functional survival in coma patients, namely prevention of secondary brain injury events. Accordingly, secondary brain injury risks associated with a cervical collar removal policy should be commensurate with the risk for CS instability. It is also important to mitigate secondary spinal injury related to CS instability.

### Risk for neurologic deficit

The CS instability rate of 2.5% is a liberal estimate from 14 relevant studies. The data are from prospective and retrospective investigations of comatose and obtunded patients without apparent spinal deficit. Operative stabilization or halo vest application indicates CS instability. Although continuation of the cervical collar for 4 to 6 weeks is suggestive of CS instability, it is questionable how many such patients really are unstable. The most relevant data are probably from the five prospective studies of purely comatose patients [3,13,21,23,29]. These investigations include 1,156 patients, with no description of any patient needing a cervical collar for 4 to 6 weeks; however, one patient had CS instability (0.1%). The recent and relevant meta-analysis by Muchow and coworkers [74] supports the effectiveness of comprehensive CS CT scanning. The authors found that MRI was not necessary to diagnose CS injuries that require surgical stabilization. The data presented in Table 1 represent evidence that may be helpful when computing the estimated CS instability rate. However, they do not represent an endorsement that plain radiographs and supplementary CT scanning is as effective as comprehensive CT scanning.

In comatose patients with no apparent spinal deficit and a negative comprehensive CS CT scan, it is reasonable to estimate that 11 functional survivors per 1,000 comatose patients will have isolated CS instability (literature-based functional survivor rate is 44.4% and CS instability rate is 2.5%). With cervical collar removal and omission of a CS MRI, there is no certainty regarding how many patients will develop a spinal neurologic deficit and convert to death or severe disability status. None, all, or a proportion of these patients may develop a severe spinal cord deficit. Levi and colleagues [75] recently reviewed data from eight level I trauma centers to characterize patients developing spinal neurologic deterioration after arrival at the trauma center. The target group consists of patients with neurologic decline because of an unrecognized fracture, subluxation, or soft tissue injury of the cervical, thoracic, or lumbar spine. The 24 patients represent 0.21% of spine fracture or strain patients and 0.025% of all trauma patients. Extrapolated, this is 1 in 500 patients with spinal injury or 1 in 4,000 trauma patients. All 24 patients had fractures or dislocations. Only two patients were comatose and no patient had an isolated ligamentous injury with normal bony spinal column alignment. Most patients had inadequate radiographic imaging. Other diagnostic problems were radiographic misinterpretations and poor-quality radiographs. That study, in addition to our literature review, suggests that isolated ligamentous injury causing subsequent neurologic deficit is uncommon and that the primary problem is inadequate imaging.

### Risks associated with cervical collar use

It is clear that cervical collar application often increases ICP with traumatic brain injury. This creates a substantial risk for secondary brain injury in those with recalcitrant intracranial hypertension. Although less concerning in patients without refractory intracranial hypertension, the collar can raise the ICP to 20 mmHg or greater in a tangible number. There may be little harm from this, if ICP is normal. Adding to this evidence are other studies that found an increase in cerebral spinal pressure with cervical collar application in non-brain-injured patients [76,77].

The study conducted by Stelfox and coworkers [18] indicated that ICU complications (for example, pressure ulcers, delirium, and VAP) increase with prolonged cervical collar use. Late cervical collar removal has an association with 1 more day on the ventilator and an added day in the ICU. The authors concluded that outcomes improve with early collar removal in obtunded trauma patients with no apparent spinal deficit and a negative CS CT.

For unstable patients, the expected functional survivor rate is 35% (350 per 1,000 patients) and the expected death or severe disability rate is 65% (650 per 1,000 patients). Accordingly, there would be nine expected functional survivors in 1,000 comatose unstable patients with CS instability. Thus, the cervical collar would be unnecessary in 991 of the 1,000 unstable patients. Likewise, the cervical collar would be unnecessary in 996 and 985 of the comatose high-risk and stable patient groups, respectively. Other investigators also recommended early collar removal because of the plethora of complications seen with prolonged use in unconscious trauma patients [11]. More specifically, several investigators have described serious skin ulceration with prolonged cervical collar use [11,78-81].

The effectiveness of CS immobilization with a cervical collar is in doubt. Restriction of CS motion varies among commonly used trauma patient immobilization devices [82-86]. Although these appliances are restrictive, substantial CS motion can occur [84,87]. Other investigators question the capacity of commonly used devices to immobilize an unstable CS [88,89]. Some utilize the cervical collar as a reminder the CS 'is not cleared'. However, this seems rather a risky and expensive 'sticky note'.

### Risks associated with MRI

Our literature review indicates that transportation of critically ill neurologic patients out of the ICU is an important risk factor for secondary brain injury. Technical mishaps also take place in this cohort [33,35,36]. Supporting this concern is the documentation of physiologic deterioration and mechanical misadventures in non-neurologic critically ill patients [90-94]. The likelihood of incurring a technical mishap or instability during out-of-ICU transportation varies with severity of illness in neurologic and non-neurologic patients [33,37,39,91,93,95]. Although stable patients are less likely to be at risk during out-of-ICU transportation, secondary brain injury risk remains an important matter. The recent meta-analysis conducted by Muchow and coworkers [74] highlighted concerns with use of MRI for surveillance: 'No adverse effects of MRI were discussed in the studies reviewed in this meta-analysis. However, MRI in an obtunded patient is associated with numerous logistical difficulties. Although in the scanner, patients may not be adequately monitored, and because of long scan times may miss other interventions. These factors are difficult to quantify but should be considered when using this technology for screening purposes.'

Performing a CS MRI requires supine positioning of the comatose brain-injured patient. The present literature review shows that ICP is likely to increase, and there is a substantial risk for aspiration or VAP in this position. The cited literature indicates that aspiration can occur within 30 to 60 minutes in mechanically ventilated patients, despite endotracheal tube cuff inflation.

The literature is replete with documentation of MRI environmental complications in critically ill patients. Technical hazards, limitations in routine ICU monitoring, and prohibition or needed alterations in common ICU therapies create risks for physiologic instability and morbidity [96-99]. Adding to this evidence, the Joint Commission issued a Sentinel Event Alert in February 2008, entitled 'Preventing accidents and injuries in the MRI suite' [100].

The MRI environment is not conducive to routine ICU monitoring and therapy, thus placing critically ill patients at risk. Studies regarding feasibility of MRI for assessing patients with acute stroke described medical instability as a contraindication or a major limitation of the MRI environment [101-103]. One acute stroke MRI feasibility study [103] described hypoxia as a frequent event, and pulse oximetry monitoring was often not possible because of patient agitation or poor peripheral perfusion.

With severe brain injury, there is commonly a need for sedation to control ICP and prevent ventilator asynchrony [104-106]. Of concern, withdrawal of sedation has an untoward effect in severe brain injury [107]. Continued ICU sedation during MRI scanning prevents movement that can affect image quality, create ventilator asynchrony, and raise ICP. Traumatic brain injury sedation is often by continuous infusion [108-111]. However, maintenance of sedative infusions in the MRI environment is complex. This challenge occurs in a setting that limits physiologic monitoring and visualization.

### Brain injury severity: assessment of risks associated with cervical collar use and MRI

The cervical collar and CS MRI create potential risks in unstable patients. Risks associated with cervical collar use include those for intracranial hypertension and increased ICU complications. MRI risks include intracranial hypertension, systemic hypotension, hypoxia, and aspiration or VAP. Numerical estimates of cervical collar and MRI risks indicate a 6-fold to 29-fold increase in comparison with the CS instability estimate. Cervical collar and CS MRI cause potential risks in high-risk patients. The risk with cervical collar use is for increased ICU complications, and risks with MRI include those for systemic hypotension, hypoxia, and aspiration or VAP. The numerical estimates of cervical collar and MRI risks indicate a 6-fold to 10-fold increase in comparison with the CS instability estimate. Cervical collar and CS MRI carry potential risks in stable patients. The risk with cervical collar use is for increased ICU complications, and risks with MRI include those for systemic hypotension, hypoxia, and aspiration or VAP. The numerical estimates of cervical collar and MRI risks indicate a 4-fold to 10-fold increase in comparison with the CS instability estimate.

Literature documentation shows that VAP increases death and severe disability in severe brain-injured patients. Prolonged cervical collar use increases ICU complications in obtunded patients, but it does not appear to worsen brain injury outcomes. The effect on severely brain-injured patients is uncertain. Early collar removal without MRI appears appropriate for most unstable patients. Early collar removal without MRI may be fitting for most high-risk patients. Early MRI may be reasonable in stable patients. However, early collar removal without MRI may be a lower risk strategy for selected complex patients. With prolonged cervical collar use or performance of screening CS MRI, the neurosurgeon, trauma surgeon, and intensivist should ponder the notion that the quest to prevent secondary spinal injury may actually prevent potential brain-injured functional survivors from realizing their destiny.

### Costs of prolonged cervical collar use and MRI scanning

Stelfox and coworkers [18] demonstrated that prolonged cervical collar use is associated with an extra day on the ventilator and an additional day in the ICU. The cost estimate for 1 day of mechanical ventilation per patient (in US$) is $2,192 [112]. The approximate cost for 1 day of nonventilator ICU care per patient is $1,521 [112]. The ICU complication cost estimate for prolonged cervical collar use intending to protect one to three patients in 1,000 is $3.7 million ([$2,192 + $1,521] × 1,000 patients). The charge for a cervical spine MRI is approximately $2,000 in the authors' hospital. The MRI cost estimate to detect CS instability in one patient is $2.0 million ($2,000 × 1,000 patients). Early cervical collar removal without an MRI mitigates the estimated $5.7 million expense.

BlueCross BlueShield Association has concerns about escalating costs associated with the rapid growth of diagnostic imaging [113]. The Association is exploring ways to promote the safe, effective, and efficient provision of imaging services. Although there is no relevant cost-effectiveness analysis, such an investigation would be valuable. The analysis should include expenses for potential functional survivors who acquire severe disability from secondary spine injury, associated with early collar removal without CS MRI. It should also include costs for potential functional survivors who incur severe disability from secondary brain injury, related to cervical collar use and CS MRI.

### CS CT assessment

It has been recommended that CS CT scanning include occiput through the first thoracic vertebra and consist of axial views with coronal and sagittal reformats [20,22,23,27,114-116]. We recommend a formal institutional review for comatose patients with a negative CS CT evaluation. The findings of the study conducted by Levi and coworkers [75] support this notion. We suggest that two physicians review the scan, for instance radiologist, trauma surgeon, and/or spine surgeon. Medical record documentation should include the date and time of the physicians' review. The note ought to describe the visual quality of the scan (adequate) and pertinent findings: no fracture, no CS malalignment, and no excessive prevertebral body swelling.

### Pre-existing CS disease

Some investigators have identified an increased CS fracture rate in the elderly [117], whereas others have not [118]. However, the elderly often have pre-existing degenerative CS disease [119,120]. To our knowledge, there are no data describing comatose patients with pre-existing CS disease to determine whether their risk for isolated ligamentous instability is different from the risks cited here. Levi and coworkers [75] implied that superimposed degenerative changes may cause the diagnosis of spinal injury to be more complex. Therefore, our conclusions may not be applicable to patients with pre-existing CS pathology – degenerative or otherwise.

### Follow-up in patients with early cervical collar removal without MRI

Should the clinician decide that it is best to remove the cervical collar without MRI, it is prudent to evaluate these patients for evidence of CS instability. Appropriate follow up includes a daily extremity motor examination and an evaluation for neck pain or tenderness when the patient becomes vigilant. Subsequent radiographs could include a lateral cervical spine radiography or, should a repeat brain CT be necessary, a CS CT. Other options might include replacement of the cervical collar or obtaining CS MRI after the patient is more stable.

### Study limitations

There are constraints in precisely delineating the rate of CS instability. There may be a bias in the literature, with failure to report patients with missed CS instability and development of neurologic deficit. However, the low CS instability rate is probably representative because the estimate comes from 14 prospective and retrospective studies that include 2,000 patients with a confirmatory evaluation. The study by Levi and coworkers [75] is supportive.

The literature does not precisely define the risk for CS instability with a negative CT scan when pre-existing CS disease is present. The precise risk for death or severe disability with missed CS instability, out-of-ICU scans, and prolonged cervical collar use, beyond their severe brain injury risk, is uncertain. However, the secondary brain injury risks are likely to be tangible. A randomized controlled trial of early collar removal with and without MRI in stable comatose patients might be edifying.

## Conclusion

The death or severe disability rate for blunt trauma coma is formidable and relates to brain injury severity. The principal brain salvage objective is to avoid secondary brain injury events. Because CS injury is substantial with coma, prevention of secondary spine injury is also important. A comprehensive CS CT (occiput to T-1 axial views, with sagittal and coronal reformats) with the first brain CT is prudent. When there is no apparent spinal deficit and comprehensive CT reveals no fracture, malalignment, or prevertebral swelling, the CS instability rate is 2.5%. A formal CS CT review should confirm that no sign of acute bony injury (no fracture, malalignment, or prevertebral swelling) exists. The 2.5% CS instability rate may not be accurate in patients with pre-existing CS disease. Cervical collar use and CS assessment with MRI carry risks for secondary brain injury, in part related to brain injury severity. The literature-based evidence suggests that prolonged cervical collar use and MRI secondary brain injury risks are more likely than CS instability. The objective of clinical decision-making is to minimize secondary brain injury and secondary spinal injury risks. Brain injury severity (unstable, high risk, or stable), probability of CS instability, cervical collar risk, and MRI risk assessments are essential for deciding whether CS MRI is appropriate and determining the optimal timing of cervical collar removal. This review suggests that early collar removal without MRI may be a lower risk strategy for some comatose patients with negative comprehensive CS CT and no apparent spinal deficit, when compared with prolonged collar use or CS MRI.

## Key messages

• Blunt trauma coma functional survivor outcomes (capable of independent activities of daily living) are poor

• When comprehensive CS CT is negative and there is no apparent spinal deficit, CS instability is unlikely (2.5%).

• Secondary brain injury associated with a cervical collar or CS MRI is more probable than CS instability and may jeopardize cerebral recovery.

• Brain injury severity (unstable, high risk, or stable), probability of CS instability, cervical collar risk, and MRI risk assessments are essential for deciding whether CS MRI is appropriate and determining the optimal timing of cervical collar removal.

• For some comatose patients with negative comprehensive CS CT and no apparent spinal deficit, early collar removal without CS MRI may be appropriate.

• After early collar removal without MRI, daily examination of the patient for CS instability is prudent.

## Abbreviations

CI = confidence interval; CS = cervical spine; CT = computed tomography; GCS = Glasgow Coma Scale; ICP = intracranial pressure; ICU = intensive care unit; MeSH = medical subject heading; MRI = magnetic resonance imaging; VAP = ventilator-associated pneumonia.

## Competing interests

The authors declare that they have no competing interests.

## Authors' contributions

Three times, CMD has been a member of an Eastern Association for the Surgery of Trauma committee charged with developing guidelines for evaluating the cervical spine after traumatic injury. CMD formed a multidisciplinary committee to review the literature regarding negative cervical spine imaging in comatose brain-injured patients: trauma surgeon and surgical intensivist (CMD), neurosurgeon (BPB), radiologist (BDC), and medical research director (DJG). CMD, BPB, BDC, and DJG developed the relevant hypotheses. CMD, BPB, BDC, and DJG formulated the relevant risks and determined the germane literature. CMD conducted the literature search. CMD, BPB, BDC, and DJG summarized the literature findings. CMD, BPB, BDC, and DJG determined how to represent the relevant risks. CMD, BPB, BDC, and DJG developed, reviewed, and refined several manuscript drafts. CMD, BPB, BDC, and DJG formulated the final manuscript.

## References

[B1] Barba CA, Taggert J, Morgan AS, Guerra J, Bernstein B, Lorenzo M, Gershon A, Epstein N (2001). A new cervical spine clearance protocol using computed tomography. J Trauma.

[B2] Chiu WC, Haan JM, Cushing BM, Kramer ME, Scalea TM (2001). Ligamentous injuries of the cervical spine in unreliable blunt trauma patients: incidence, evaluation, and outcome. J Trauma.

[B3] Brohi K, Healy M, Fotheringham T, Chan O, Aylwin C, Whitley S, Walsh M (2005). Helical computed tomographic scanning for the evaluation of the cervical spine in the unconscious, intubated trauma patient. J Trauma.

[B4] Brooks RA, Willett KM (2001). Evaluation of the Oxford protocol for total spinal clearance in the unconscious trauma patient. J Trauma.

[B5] Daffner RH (2004). Controversies in cervical spine imaging in trauma patients. Emerg Radiol.

[B6] Blackmore CC, Ramsey SD, Mann FA, Deyo RA (1999). Cervical spine screening with CT in trauma patients: a cost-effectiveness analysis. Radiology.

[B7] Blackmore CC, Mann FA, Wilson AJ (2000). Helical CT in the primary trauma evaluation of the cervical spine: an evidence-based approach. Skeletal Radiol.

[B8] Jones PS, Wadley J, Healy M (2004). Clearing the cervical spine in unconscious adult trauma patients: a survey of practice in specialist centres in the UK. Anaesthesia.

[B9] Tins B, Cassar-Pullicino V (2007). Controversies in 'clearing' trauma to the cervical spine. Semin Ultrasound CT MR.

[B10] Weinberg L, Hiew CY, Brown DJ, Lim EJ, Hart GK (2007). Isolated ligamentous cervical spinal injury in the polytrauma patient with a head injury. Anaesth Intensive Care.

[B11] Morris CG, McCoy EP, Lavery GG (2004). Spinal immobilisation for unconscious patients with multiple injuries. BMJ.

[B12] Sliker CW, Mirvis SE, Shanmuganathan K (2005). Assessing cervical spine stability in obtunded blunt trauma patients: review of medical literature. Radiology.

[B13] Davis JW, Kaups KL, Cunningham MA, Parks SN, Nowak TP, Bilello JF, Williams JL (2001). Routine evaluation of the cervical spine in head-injured patients with dynamic fluoroscopy: a reappraisal. J Trauma.

[B14] Bolinger B, Shartz M, Marion D (2004). Bedside fluoroscopic flexion and extension cervical spine radiographs for clearance of the cervical spine in comatose trauma patients. J Trauma.

[B15] Ajani AE, Cooper DJ, Scheinkestel CD, Laidlaw J, Tuxen DV (1998). Optimal assessment of cervical spine trauma in critically ill patients: a prospective evaluation. Anaesth Intensive Care.

[B16] Griffiths HJ, Wagner J, Anglen J, Bunn P, Metzler M (2002). The use of forced flexion/extension views in the obtunded trauma patient. Skeletal Radiol.

[B17] Sees DW, Rodriguez Cruz LR, Flaherty SF, Ciceri DP (1998). The use of bedside fluoroscopy to evaluate the cervical spine in obtunded trauma patients. J Trauma.

[B18] Stelfox HT, Velmahos GC, Gettings E, Bigatello LM, Schmidt U (2007). Computed tomography for early and safe discontinuation of cervical spine immobilization in obtunded multiply injured patients. J Trauma.

[B19] D'Alise MD, Benzel EC, Hart BL (1999). Magnetic resonance imaging evaluation of the cervical spine in the comatose or obtunded trauma patient. J Neurosurg.

[B20] Hogan GJ, Mirvis SE, Shanmuganathan K, Scalea TM (2005). Exclusion of unstable cervical spine injury in obtunded patients with blunt trauma: is MR imaging needed when multi-detector row CT findings are normal?. Radiology.

[B21] Padayachee L, Cooper DJ, Irons S, Ackland HM, Thomson K, Rosenfeld J, Kossmann T (2006). Cervical spine clearance in unconscious traumatic brain injury patients: dynamic flexion-extension fluoroscopy versus computed tomography with three-dimensional reconstruction. J Trauma.

[B22] Adams JM, Cockburn MI, Difazio LT, Garcia FA, Siegel BK, Bilaniuk JW (2006). Spinal clearance in the difficult trauma patient: a role for screening MRI of the spine. Am Surg.

[B23] Como JJ, Thompson MA, Anderson JS, Shah RR, Claridge JA, Yowler CJ, Malangoni MA (2007). Is magnetic resonance imaging essential in clearing the cervical spine in obtunded patients with blunt trauma?. J Trauma.

[B24] Ghanta MK, Smith LM, Polin RS, Marr AB, Spires WV (2002). An analysis of Eastern Association for the Surgery of Trauma practice guidelines for cervical spine evaluation in a series of patients with multiple imaging techniques. Am Surg.

[B25] Menaker J, Philp A, Boswell S, Scalea TM (2008). Computed tomography alone for cervical spine clearance in the unreliable patient: are we there yet?. J Trauma.

[B26] Sarani B, Waring S, Sonnad S, Schwab CW (2007). Magnetic resonance imaging is a useful adjunct in the evaluation of the cervical spine of injured patients. J Trauma.

[B27] Schuster R, Waxman K, Sanchez B, Becerra S, Chung R, Conner S, Jones T (2005). Magnetic resonance imaging is not needed to clear cervical spines in blunt trauma patients with normal computed tomographic results and no motor deficits. Arch Surg.

[B28] Stassen NA, Williams VA, Gestring ML, Cheng JD, Bankey PE (2006). Magnetic resonance imaging in combination with helical computed tomography provides a safe and efficient method of cervical spine clearance in the obtunded trauma patient. J Trauma.

[B29] Widder S, Doig C, Burrowes P, Larsen G, Hurlbert RJ, Kortbeek JB (2004). Prospective evaluation of computed tomographic scanning for the spinal clearance of obtunded trauma patients: preliminary results. J Trauma.

[B30] Mobbs RJ, Stoodley MA, Fuller J (2002). Effect of cervical hard collar on intracranial pressure after head injury. ANZ J Surg.

[B31] Davies G, Deakin C, Wilson A (1996). The effect of a rigid collar on intracranial pressure. Injury.

[B32] Hunt K, Hallworth S, Smith M (2001). The effects of rigid collar placement on intracranial and cerebral perfusion pressures. Anaesthesia.

[B33] Gunnarsson T, Theodorsson A, Karlsson P, Fridriksson S, Boström S, Persliden J, Johansson I, Hillman J (2000). Mobile computerized tomography scanning in the neurosurgery intensive care unit: increase in patient safety and reduction of staff workload. J Neurosurg.

[B34] Peerdeman SM, Girbes AR, Vandertop WP (2002). Changes in cerebral glycolytic activity during transport of critically ill neurotrauma patients measured with microdialysis. J Neurol.

[B35] Doring BL, Kerr ME, Lovasik DA, Thayer T (1999). Factors that contribute to complications during intrahospital transport of the critically ill. J Neurosci Nurs.

[B36] Kondo K, Herman SD, O'Reilly LP, Simeonidis S (1985). Transport system for critically ill patients. Crit Care Med.

[B37] Kalisch BJ, Kalisch PA, Burns SM, Kocan MJ, Prendergast V (1995). Intrahospital transport of neuro ICU patients. J Neurosci Nurs.

[B38] Bekar A, Ipekoglu Z, Tureyen K, Bilgin H, Korfali G, Korfali E (1998). Secondary insults during intrahospital transport of neurosurgical intensive care patients. Neurosurg Rev.

[B39] Andrews PJD, Piper IR, Dearden NM, Miller JD (1990). Secondary insults during intrahospital transport of head-injured patients. Lancet.

[B40] Ng I, Lim J, Wong HB (2004). Effects of head posture on cerebral hemodynamics: its influences on intracranial pressure, cerebral perfusion pressure, and cerebral oxygenation. Neurosurgery.

[B41] Winkelman C (2000). Effect of backrest position on intracranial and cerebral perfusion pressures in traumatically brain-injured adults. Am J Crit Care.

[B42] Schneider GH, von Helden GH, Franke R, Lanksch WR, Unterberg A (1993). Influence of body position on jugular venous oxygen saturation, intracranial pressure and cerebral perfusion pressure. Acta Neurochir Suppl (Wien).

[B43] Durward QJ, Amacher AL, Del Maestro RF, Sibbald WJ (1983). Cerebral and cardiovascular responses to changes in head elevation in patients with intracranial hypertension. J Neurosurg.

[B44] Kiening KL, Hartl R, Unterberg AW, Schneider GH, Bardt T, Lanksch WR (1997). Brain tissue pO2-monitoring in comatose patients: implications for therapy. Neurol Res.

[B45] Meixensberger J, Baunach S, Amschler J, Dings J, Roosen K (1997). Influence of body position on tissue-pO2, cerebral perfusion pressure and intracranial pressure in patients with acute brain injury. Neurol Res.

[B46] Lee ST (1989). Intracranial pressure changes during positioning of patients with severe head injury. Heart Lung.

[B47] March K, Mitchell P, Grady S, Winn R (1990). Effect of backrest position on intracranial and cerebral perfusion pressures. J Neurosci Nurs.

[B48] Piek J, Chesnut RM, Marshall LF, van Berkum-Clark M, Klauber MR, Blunt BA, Eisenberg HM, Jane JA, Marmarou A, Foulkes MA (1992). Extracranial complications of severe head injury. J Neurosurg.

[B49] Hsieh AH, Bishop MJ, Kubilis PS, Newell DW, Pierson DJ (1992). Pneumonia following closed head injury. Am Rev Respir Dis.

[B50] Woratyla SP, Morgan AS, Mackay L, Bernstein B, Barba C (1995). Factors associated with early onset pneumonia in the severely brain-injured patient. Conn Med.

[B51] Cazzadori A, Di Perri G, Vento S, Bonora S, Fendt D, Rossi M, Lanzafame M, Mirandola F, Concia E (1997). Aetiology of pneumonia following isolated closed head injury. Respir Med.

[B52] Zygun DA, Zuege DJ, Boiteau PJ, Laupland KB, Henderson EA, Kortbeek JB, Doig CJ (2006). Ventilator-associated pneumonia in severe traumatic brain injury. Neurocrit Care.

[B53] Ferrer R, Artigas A (2002). Clinical review: non-antibiotic strategies for preventing ventilator-associated pneumonia. Crit Care.

[B54] Keenan SP, Heyland DK, Jacka MJ, Cook D, Dodek P (2002). Ventilator-associated pneumonia. Prevention, diagnosis, and therapy. Crit Care Clin.

[B55] Koeman M, Ven AJ van der, Ramsay G, Hoepelman IM, Bonten MJ (2001). Ventilator-associated pneumonia: recent issues on pathogenesis, prevention and diagnosis. J Hosp Infect.

[B56] Ewig S, Torres A (2002). Prevention and management of ventilator-associated pneumonia. Curr Opin Crit Care.

[B57] Fernandez-Crehuet R, Diaz-Molina C, de Irala J, Martinez-Concha D, Salcedo-Leal I, Masa-Calles J (1997). Nosocomial infection in an intensive-care unit: identification of risk factors. Infect Control Hosp Epidemiol.

[B58] Torres A, Serra-Batlles J, Ros E, Piera C, Puig de la Bellacasa J, Cobos A, Lomeña F, Rodríguez-Roisin R (1992). Pulmonary aspiration of gastric contents in patients receiving mechanical ventilation: the effect of body position. Ann Intern Med.

[B59] Drakulovic MB, Torres A, Bauer TT, Nicolas JM, Nogue S, Ferrer M (1999). Supine body position as a risk factor for nosocomial pneumonia in mechanically ventilated patients: a randomised trial. Lancet.

[B60] Kollef MH (1993). Ventilator-associated pneumonia. A multivariate analysis. JAMA.

[B61] Marshall LF, Gautille T, Klauber MR, Eisenberg HM, Jane JA, Luerssen TG, Marmarou A, Foulkes MA (1991). The outcome of severe closed head injury. J Neurosurg.

[B62] Fearnside MR, Cook RJ, McDougall P, McNeil RJ (1993). The Westmead Head Injury Project outcome in severe head injury. A comparative analysis of pre-hospital, clinical and CT variables. Br J Neurosurg.

[B63] Schreiber MA, Aoki N, Scott BG, Beck JR (2002). Determinants of mortality in patients with severe blunt head injury. Arch Surg.

[B64] Jiang JY, Gao GY, Li WP, Yu MK, Zhu C (2002). Early indicators of prognosis in 846 cases of severe traumatic brain injury. J Neurotrauma.

[B65] Lannoo E, Van Rietvelde F, Colardyn F, Lemmerling M, Vandekerckhove T, Jannes C, De Soete G (2000). Early predictors of mortality and morbidity after severe closed head injury. J Neurotrauma.

[B66] Juul N, Morris GF, Marshall SB, Marshall LF (2000). Intracranial hypertension and cerebral perfusion pressure: influence on neurological deterioration and outcome in severe head injury. The Executive Committee of the International Selfotel Trial. J Neurosurg.

[B67] Chesnut RM, Marshall LF, Klauber MR, Blunt BA, Baldwin N, Eisenberg HM, Jane JA, Marmarou A, Foulkes MA (1993). The role of secondary brain injury in determining outcome from severe head injury. J Trauma.

[B68] Berrouane Y, Daudenthun I, Riegel B, Emery MN, Martin G, Krivosic R, Grandbastien B (1998). Early onset pneumonia in neurosurgical intensive care unit patients. J Hosp Infect.

[B69] Cortinas SM, Lizan GM, Jimenez-Vizuete JM, Moreno CJ, Cuesta GJ, Peyro GR (2007). Incidences of early- and late-onset ventilator-associated pneumonia in a postanesthesia and critical care unit [in Spanish]. Rev Esp Anestesiol Reanim.

[B70] Bronchard R, Albaladejo P, Brezac G, Geffroy A, Seince PF, Morris W, Branger C, Marty J (2004). Early onset pneumonia: risk factors and consequences in head trauma patients. Anesthesiology.

[B71] Leone M, Bourgoin A, Giuly E, Antonini F, Dubuc M, Viviand X, Albanèse J, Martin C (2002). Influence on outcome of ventilator-associated pneumonia in multiple trauma patients with head trauma treated with selected digestive decontamination. Crit Care Med.

[B72] Kallel H, Chelly H, Bahloul M, Ksibi H, Dammak H, Chaari A, Ben Hamida C, Rekik N, Bouaziz M (2005). The effect of ventilator-associated pneumonia on the prognosis of head trauma patients. J Trauma.

[B73] Vollmer DG, Torner JC, Jane JA, Sadovnic B, Charlebois D, Eisenberg HM, Foulkes MA, Marmarou A, Marshall LF (1991). Age and outcome following traumatic coma: why do older patients fare worse?. J Neurosurg.

[B74] Muchow RD, Resnick DK, Abdel MP, Munoz A, Anderson PA (2008). Magnetic resonance imaging (MRI) in the clearance of the cervical spine in blunt trauma: A meta-analysis. J Trauma.

[B75] Levi AD, Hurlbert RJ, Anderson P, Fehlings M, Rampersaud R, Massicotte EM, France JC, Le Huec JC, Hedlund R, Arnold P (2006). Neurologic deterioration seconday to unrecognized spinal instability following trauma: a multicenter study. Spine.

[B76] Kolb JC, Summers RL, Galli RL (1999). Cervical collar-induced changes in intracranial pressure. Am J Emerg Med.

[B77] Raphael JH, Chotai R (1994). Effects of the cervical collar on cerebrospinal fluid pressure. Anaesthesia.

[B78] Hogan BJ, Blaylock B, Tobian TL (1997). Trauma multidisciplinary QI project: evaluation of cervical spine clearance, collar selection, and skin care. J Trauma Nurs.

[B79] Krock N (1997). Immobilizing the cervical spine using a collar. Complications and nursing management. Axone.

[B80] Ackland HM, Cooper DJ, Malham GM, Kossmann T (2007). Factors predicting cervical collar-related decubitus ulceration in major trauma patients. Spine.

[B81] Hewitt S (1994). Skin necrosis caused by a semi-rigid cervical collar in a ventilated patient with multiple injuries. Injury.

[B82] McCabe JB, Nolan DJ (1986). Comparison of the effectiveness of different cervical immobilization collars. Ann Emerg Med.

[B83] Rosen PB, McSwain NE, Arata M, Stahl S, Mercer D (1992). Comparison of two new immobilization collars. Ann Emerg Med.

[B84] Sandler AJ, Dvorak J, Humke T, Grob D, Daniels W (1996). The effectiveness of various cervical orthoses. An in vivo comparison of the mechanical stability provided by several widely used models. Spine.

[B85] Askins V, Eismont FJ (1997). Efficacy of five cervical orthoses in restricting cervical motion. A comparison study. Spine.

[B86] Zhang S, Wortley M, Clowers K, Krusenklaus JH (2005). Evaluation of efficacy and 3D kinematic characteristics of cervical orthoses. Clin Biomech (Bristol, Avon).

[B87] Hughes SJ (1998). How effective is the Newport/Aspen collar? A prospective radiographic evaluation in healthy adult volunteers. J Trauma.

[B88] McGuire RA, Degnan G, Amundson GM (1990). Evaluation of current extrication orthoses in immobilization of the unstable cervical spine. Spine.

[B89] Richter D, Latta LL, Milne EL, Varkarakis GM, Biedermann L, Ekkernkamp A, Ostermann PA (2001). The stabilizing effects of different orthoses in the intact and unstable upper cervical spine: a cadaver study. J Trauma.

[B90] Lovell MA, Mudaliar MY, Klineberg PL (2001). Intrahospital transport of critically ill patients: complications and difficulties. Anaesth Intensive Care.

[B91] Lahner D, Nikolic A, Marhofer P, Koinig H, Germann P, Weinstabl C, Krenn CG (2007). Incidence of complications in intrahospital transport of critically ill patients: experience in an Austrian university hospital. Wien Klin Wochenschr.

[B92] Evans A, Winslow EH (1995). Oxygen saturation and hemodynamic response in critically ill, mechanically ventilated adults during intrahospital transport. Am J Crit Care.

[B93] Waydhas C, Schneck G, Duswald KH (1995). Deterioration of respiratory function after intra-hospital transport of critically ill surgical patients. Intensive Care Med.

[B94] Indeck M, Peterson S, Smith J, Brotman S (1988). Risk, cost, and benefit of transporting ICU patients for special studies. J Trauma.

[B95] Smith I, Fleming S, Cernaianu A (1990). Mishaps during transport from the intensive care unit. Crit Care Med.

[B96] ECRI (2001). The safe use of equipment in the magnetic resonance environment. Health Devices.

[B97] ECRI (2005). What's new in MR safety: the latest on the safe use of equipment in the magnetic resonance environment. Health Devices.

[B98] Soulen RL, Duman RJ, Hoeffner E (1994). Magnetic resonance imaging in the critical care setting. Crit Care Clin.

[B99] Ewart MC (2003). MRI and its dangers for critically ill patients. Care of the Critically Ill.

[B100] Joint Commission Sentinel Event Alert: Preventing accidents and injuries in the MRI suite. http://www.jointcommission.org/SentinelEvents/SentinelEventAlert/sea_38.htm.

[B101] Singer OC, Sitzer M, du Mesnil dR, Neumann-Haefelin T (2004). Practical limitations of acute stroke MRI due to patient-related problems. Neurology.

[B102] Tan PL, King D, Durkin CJ, Meagher TM, Briley D (2006). Diffusion weighted magnetic resonance imaging for acute stroke: practical and popular. Postgrad Med J.

[B103] Hand PJ, Wardlaw JM, Rowat AM, Haisma JA, Lindley RI, Dennis MS (2005). Magnetic resonance brain imaging in patients with acute stroke: feasibility and patient related difficulties. J Neurol Neurosurg Psychiatry.

[B104] Brain Trauma Foundation, American Association of Neurological Surgeons, Congress of Neurological Surgeons (2007). Guidelines for the management of severe traumatic brain injury. XI. Anesthetics, analgesics, and sedatives. J Neurotrauma.

[B105] Prielipp RC, Coursin DB (1995). Sedative and neuromuscular blocking drug use in critically ill patients with head injuries. New Horiz.

[B106] Hsiang JK, Chesnut RM, Crisp CB, Klauber MR, Blunt BA, Marshall LF (1994). Early, routine paralysis for intracranial pressure control in severe head injury: is it necessary?. Crit Care Med.

[B107] Bruder N, Lassegue D, Pelissier D, Graziani N, Francois G (1994). Energy expenditure and withdrawal of sedation in severe head-injured patients. Crit Care Med.

[B108] Sanchez-Izquierdo-Riera JA, Caballero-Cubedo RE, Perez-Vela JL, Ambros-Checa A, Cantalapiedra-Santiago JA, Alted-Lopez E (1998). Propofol versus midazolam: safety and efficacy for sedating the severe trauma patient. Anesth Analg.

[B109] Kelly DF, Goodale DB, Williams J, Herr DL, Chappell ET, Rosner MJ, Jacobson J, Levy ML, Croce MA, Maniker AH, Fulda GJ, Lovett JV, Mohan O, Narayan RK (1999). Propofol in the treatment of moderate and severe head injury: a randomized, prospective double-blinded pilot trial. J Neurosurg.

[B110] Bourgoin A, Albanese J, Wereszczynski N, Charbit M, Vialet R, Martin C (2003). Safety of sedation with ketamine in severe head injury patients: comparison with sufentanil. Crit Care Med.

[B111] Werner C, Kochs E, Bause H, Hoffman WE, Schulte am EJ (1995). Effects of sufentanil on cerebral hemodynamics and intracranial pressure in patients with brain injury. Anesthesiology.

[B112] Dasta JF, McLaughlin TP, Mody SH, Piech CT (2005). Daily cost of an intensive care unit day: the contribution of mechanical ventilation. Crit Care Med.

[B113] Rothenberg BM, Korn A (2005). The opportunities and challenges posed by the rapid growth of diagnostic imaging. J Am Coll Radiol.

[B114] Holmes JF, Mirvis SE, Panacek EA, Hoffman JR, Mower WR, Velmahos GC (2002). Variability in computed tomography and magnetic resonance imaging in patients with cervical spine injuries. J Trauma.

[B115] Rydberg J, Buckwalter KA, Caldemeyer KS, Phillips MD, Conces DJ, Aisen AM, Persohn SA, Kopecky KK (2000). Multisection CT: scanning techniques and clinical applications. Radiographics.

[B116] Li AE, Fishman EK (2003). Cervical spine trauma: evaluation by multidetector CT and three-dimensional volume rendering. Emerg Radiol.

[B117] Hu R, Mustard CA, Burns C (1996). Epidemiology of incident spinal fracture in a complete population. Spine.

[B118] Ong AW, Rodriguez A, Kelly R, Cortes V, Protetch J, Daffner RH (2006). Detection of cervical spine injuries in alert, asymptomatic geriatric blunt trauma patients: who benefits from radiologic imaging?. Am Surg.

[B119] Schrag SP, Toedter LJ, McQuay N (2008). Cervical spine fractures in geriatric blunt trauma patients with low-energy mechanism: are clinical predictors adequate?. Am J Surg.

[B120] Bub LD, Blackmore CC, Mann FA, Lomoschitz FM (2005). Cervical spine fractures in patients 65 years and older: a clinical prediction rule for blunt trauma. Radiology.

